# Computed Tomography Imaging under Artificial Intelligence Reconstruction Algorithm Used in Recovery of Sports Injury of the Knee Anterior Cruciate Ligament

**DOI:** 10.1155/2022/1199841

**Published:** 2022-05-28

**Authors:** Heng Zhang, Haiming Zheng, Ren Deng, Kaiwen Luo, Shukai Duan

**Affiliations:** ^1^College of Mathematics and Statistics, Southwest University, Chongqing 400715, China; ^2^Department of Military Logistics, Army Logistics Academy, Chongqing 401331, China; ^3^College of Artificial Intelligence, Southwest University, Chongqing 400715, China

## Abstract

This study aimed to analyze the influence of artificial intelligence (AI) reconstruction algorithm on computed tomography (CT) images and the application of CT image analysis in the recovery of knee anterior cruciate ligament (ACL) sports injuries. A total of 90 patients with knee trauma were selected for enhanced CT scanning and randomly divided into three groups. Group A used the filtered back projection (FBP) reconstruction algorithm, and the tube voltage was set to 120 kV during CT scanning. Group B used the iDose4 reconstruction algorithm, and the tube voltage was set to 120 kV during CT scanning. In group C, the iDose4 reconstruction algorithm was used, and the tube voltage was set to 100 kV during CT scanning. The noise, signal-to-noise ratio (SNR), carrier-to-noise ratio (CNR), CT dose index volume (CTDI), dose length product (DLP), and effective radiation dose (ED) of the three groups of CT images were compared. The results showed that the noise of groups B and C was smaller than that of group A (*P* < 0.05), and the SNR and CNR of groups B and C were higher than those of group A. The images of patients in group A with the FBP reconstruction algorithm were noisy, and the boundaries were not clear. The noise of the images obtained by the iDose4 reconstruction algorithm in groups B and C was improved, and the image resolution was also higher. The agreement between arthroscopy and CT scan results was 96%. Therefore, the iterative reconstruction algorithm of iDose4 can improve the image quality. It was of important value in the diagnosis of knee ACL sports injury.

## 1. Introduction

The anterior cruciate ligament (ACL) of the knee is a fibrous connective tissue connecting the femur and tibia, and its main function is transmitting tension and enhancing joint stability [1]. ACL injury has a high incidence in the population, and ACL fractures caused by noncontact mechanisms are common [2]. During exercise, sudden torsion, sudden stop, and weight-bearing can lead to ACL overload, resulting in ACL tear or even fracture [3]. Ligament injury can not only cause joint pain, cartilage and meniscus damage, and other soft tissue damage in the joint but may also even induce osteoarthritis if not treated in time [4]. At present, the degree of ligament relaxation, patient activity, individual clinical symptoms, and treatment strategies for injured ligaments are also different [5]. Conservative treatment mainly includes pain relief and waiting for self-healing under the protection of auxiliary devices [6]. ACL injuries mainly occur in sports and are relatively serious and difficult to treat in knee injuries [7]. It is more common than the initial belt injury of the posterior crossing, and the injury of the ACL of the knee will lead to a decrease in the stability of the knee and also accelerate the wear or injury of the articular cartilage and meniscus [8]. The fracture of the ACL inevitably has a significant impact on the stability of the knee [9]. Osteoarthritis of the knee is an aseptic and chronic arthritic disease characterized by degenerative changes in the articular cartilage and regeneration of joint margins and subchondral bones. The disease begins in the cartilage, and the degeneration of the articular cartilage is the core, which then affects the subchondral bone, synovium, joint capsule, and muscle belt. Eventually, joint pain, limited activity, and even unstable changes will be caused, thus affecting people's daily activity, ability, and quality of life [10].

Early, rapid, and accurate diagnosis of ACL injury is very important in clinical diagnosis and treatment. At present, the diagnosis of ACL injury mainly includes physical examination, such as front drawer test, axial shift test, and examination. However, in the acute phase of injury, it is affected by the patient's pain, joint swelling, and other factors that can affect the accuracy of the diagnosis. In addition, the gold standard for the diagnosis of ACL injury is arthroscopy. However, arthroscopy is not only expensive and slow but also traumatic to the patient. Using imaging can not only avoid these problems but also obtain information about the patient's knee. Computed tomography (CT) is adopted as a noninvasive imaging diagnostic method for patients with knee injuries. CT scan has the advantages of fast scanning speed, wide scanning range, and high scanning image resolution, which can provide reliable information for clinical judgment of knee injury types [11]. With the upgrading of CT in recent years, many researchers found that image reconstruction algorithms can be used to reduce the radiation risk of CT scans [12]. As artificial intelligence advances, technologies like deep learning and big data are widely used in the medical field. As a medical auxiliary diagnostic system, artificial intelligence medical imaging not only has high accuracy but also can improve the work efficiency of doctors. The traditional filtered rear projection (FBP) algorithm is very sensitive to noise and artifacts, thus limiting the reduction of the radiation dose. The iDose4 iterative reconstruction algorithm is one of the emerging computer intelligence algorithms. The introduction of the CT iDose4 iterative reconstruction algorithm can significantly optimize image quality and reduce noise while reducing the radiation dose of a CT scan [13]. Some scholars used iDose4 and FBP algorithms to process renal artery CT angiography and found that iDose4 reconstruction images were significantly better [14]. Currently, few studies applied the iDose4 iterative reconstruction algorithm to CT scan images of patients with ACL injuries of the knee joint.

This research was developed to compare the effect of original images obtained by different algorithms for patients with ACL injury of the orthopaedic knee joint with different doses of multislice spiral CT. The image quality after processing was compared with the FBP algorithm, hoping to provide a reference for the application of CT imaging in the recovery of the ACL of the knee joint.

## 2. Methods

### 2.1. Research Objects

A total of 90 patients with ACL motor injuries were recruited from September 2019 to October 2020, all of whom underwent enhanced CT scanning. Among them, 54 were male patients and 36 were female patients. All patients were between 23 and 58 years old, with an average age of 39 years. The patients were randomly rolled into groups A, B, and C. In group A, 30 patients were treated with the FBP reconstruction algorithm, and tube voltage was set to 120 kV during CT scanning. In group B, 30 patients were treated with the iDose4 reconstruction algorithm, and the tube voltage was set at 120 kV during CT scanning. In group C, 30 patients were treated with the iDose4 reconstruction algorithm, and tube voltage was set at 100 kV during CT scanning. This study had been approved by the ethics committee of the hospital, and all patients and their families had signed informed consent.

Inclusion criteria were as follows: (i) patients diagnosed with acute knee ACL sports injury according to the clinical diagnosis and treatment guidelines for ACL injury; (ii) patients who can receive a CT scan; (iii) patients without contraindications for allergy to contrast media; (iv) patients over 23 years old and younger than 58 years old; and (v) patients who signed the informed consent. Exclusion criteria were as follows: (i) patients with other concomitant fractures and knee lesions; (ii) patients with other infectious diseases or bone and joint diseases such as rheumatoid arthritis; (iii) patients suffering from serious cardiovascular and cerebrovascular diseases or liver and kidney dysfunction; (iv) patients with mental disorders; and (v) patients with communication disorders.

### 2.2. CT Scanning

The CT scanner used was a 128-slice helical CT scanner. CT scan parameter settings were given as follows: the layer thickness was 3 mm, the layer distance was also 3 mm, the rotation speed of the tube was set to 2 rpm, the pitch was 0.984, the contrast agent was iohexol, the tube current was 500 mAs, and the tube voltage was subject to the three different parameters of A, B, and C. The patient was in a supine position, and the scan range was from the distal end of the patient's femur to the proximal end of the knee joint.

### 2.3. Basic Methods of iDose4 Iterative Reconstruction Algorithm

The iDose4 iterative reconstruction algorithm belongs to a class of AI algorithms. It can not only iteratively reorganize in the projection space but also iteratively reorganize in the image space. The general flow of the iDose4 reconstruction algorithm is to reconstruct the projection data using the FBP method and construct the multinoise model and anatomical model of the obtained image data. In the process of repeated iterations, and the noise data is continuously removed to improve the image quality. Compared with the FBP algorithm, the main feature of the iDose4 algorithm is that it has a dual-space model. The iterative reconstruction expanded in the dual-space can be realized when the image texture remains unchanged, the artifacts and noise ratio of the image are reduced, and the image quality can be increased.

### 2.4. Observation Indicators

General information of patients was collected, including gender, age, body mass index (BMI), and disease location of patients in the three groups. The calculation method of BMI is shown in (1), where *W* is the weight and *H* is the height.

(1) $$\begin{array}{c} \text{BMI}=\frac{W}{H}. \end{array}$$

Subjective image rating was performed by two professional imaging physicians. The highest score was 5. A score of 1 indicated that the image quality was poor, the lesions cannot be displayed or the lesions and boundaries were not clear, and the artifacts showed great influence. A score of 2 indicated that the image quality was normal, the lesions and boundaries were blurred, but the lesions were visible. A score of 3 indicated good image quality, clear lesions and artifacts, but did not affect the diagnosis. A score of 4 indicated that the image quality was good, the influence of noise and artifacts was small, and the lesions were clear. A score of 5 indicated that the image quality was very good, without noise and artifacts, with clear lesions and clear boundaries, which can be used for diagnosis.

Two radiologists with more than three years of clinical imaging experience were invited to objectively evaluate the CT images of all patients and calculate the signal-to-noise ratio (SNR) and carrier-to-noise ratio (CNR). Three regions of interest (ROI) of the bone, adjacent muscle layer, and subcutaneous fat layer with an area of 50–100 mm^2^ were selected to calculate the mean CT values and standard deviation (SD). SD was the objective noise of the image. The smaller the SD, the less image noise and the better the image quality. The SNR and CNR calculation methods are shown in (2) and (3), where CT_a_ represents the average CT value of the bone, CT_V_ represents the average CT value of the muscle, CT_avg_ refers to the average CT value of the bone and muscle, and SD_avg_ represents the average SD value of the bone and muscle.

(2) $$\begin{array}{c} \text{SNR}=\frac{{\text{CT}}_{\text{avg}}}{{\text{SD}}_{\text{avg}}}, \end{array}$$

(3) $$\begin{array}{c} \text{CNR}=\frac{{\text{CT}}_{a}-{\text{CT}}_{v}}{{\text{SD}}_{\text{avg}}}. \end{array}$$

ACL injury in all patients was analyzed by arthroscopy and CT scan results of all patients. The radiation dose of patients was assessed by effective radiation dose (ED), CT dose index volume (CTDI), and dose length generation (DLP). The calculation method of effective radiation dose (ED) is shown in

(4) $$\begin{array}{c} \text{ED}=DL\;P\times 0.0054. \end{array}$$

The coincidence rate of CT diagnosis results and arthroscopic diagnosis results was counted. The calculation of the coincidence rate is shown in (5), where *Y* is the number of coincidences and *Z* is the total number.

(5) $$\begin{array}{c} \text{coincidence rate}=\frac{Y}{Z}. \end{array}$$

### 2.5. Statistical Analysis

All data in this study were analyzed by SPSS 20.0 statistical software; the difference between the groups was analyzed by the chi-square test method. When *P* < 0.05, it meant that the difference was statistically significant.

## 3. Results

### 3.1. Basic Information of the Two Groups of Patients

The general clinical data of all patients are shown in Table 1. The three groups of patients showed obvious differences in gender, age, body mass index (BMI), and disease site (*P* > 0.05), and they were comparable.

### 3.2. Results of Image Evaluation Indicators

#### 3.2.1. Subjective Evaluation Results

**Figure 1:**
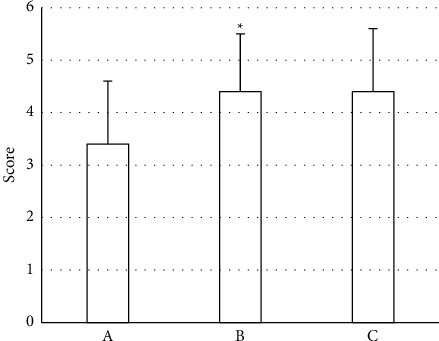
Subjective evaluation results on the image quality of the three groups of patients.*∗* indicates compared with group (A), *P* < 0.05.

As illustrated in Figure 1, the image quality scores of group B and group C were higher than those of group A, and the comparison between group B and group A was statistically obvious (*P* < 0.05). It showed that the image quality obtained by the iDose4 reconstruction algorithm was better than the image quality obtained by the FBP algorithm.

#### 3.2.2. Objective Evaluation Results

**Figure 2:**
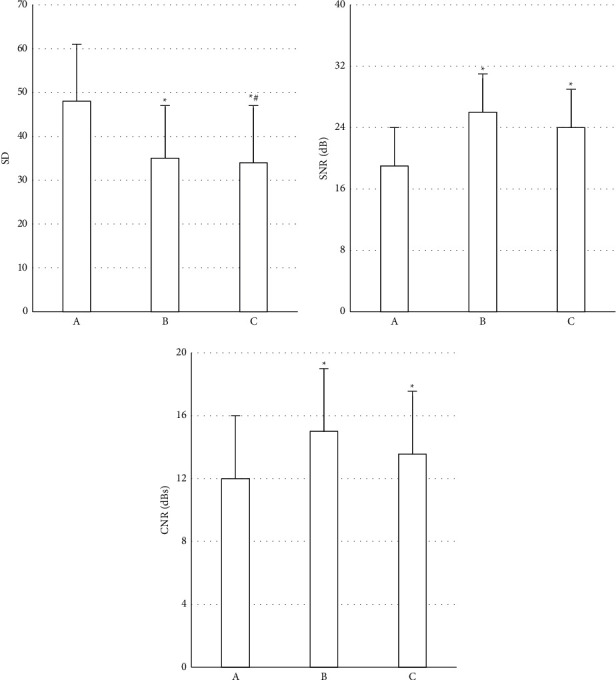
Comparison results of SD, SNR, and CNR of the three groups of patients. A is SD, B is SNR, and C is CNR. *∗* represents that the comparison difference between group A, group B, and group C, *P* < 0.05; # indicates the difference between group B and group C showed *P* < 0.05.

**Figure 3:**
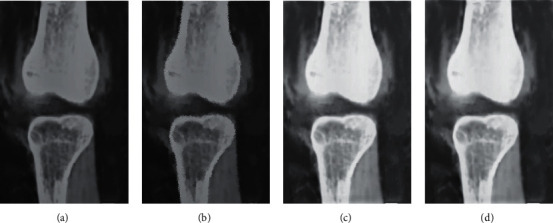
CT image of a patient with a knee ACL injury. (a) Original CT image. (b) Image processed by the FBP algorithm. (c) Image of the patient in group B. (d) Image of the patient in group C.

SD, SNR, and CNR were used as indicators for objective evaluation of the images. The result is shown in Figure 2. It was found that the noise of group B and group C was less than that of group A, which was statistically great (*P* < 0.05), and it was also statistically obvious compared with that of group B (*P* < 0.05). The SNR and CNR of groups B and C were higher than those of group A. However, the comparison of SNR and CNR between groups B and C was not statistically significant. At the same time, Figure 3 illustrates that the images of patients in group A using the FBP reconstruction algorithm were noisy, the lesions were difficult to identify, and the boundaries were not clear; while the image noise obtained by the iDose4 reconstruction algorithm in groups B and C was improved, and the image resolution was improved.

### 3.3. Evaluation of Results of Radiation Dose

**Figure 4:**
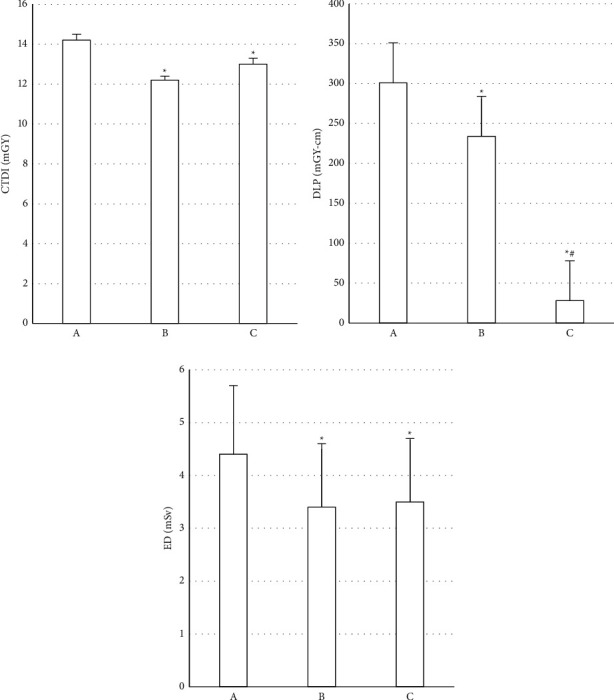
Comparison of radiation dose-related indicators among the three groups of patients. (a) CTD; (b) DLP; (c) ED. *∗* represents that the comparison difference between group A, group B, and group C *P* < 0.05; # indicates the difference between group B and group C, *P* < 0.05.

The evaluation results of the three groups of radiation doses are shown in Figure 4. Compared with group A, the three radiation dose parameters of groups B and C were effectively reduced (*P* < 0.05). The EDs of group A, group B, and group C were 4.46 ± 1.21, 3.39 ± 1.02, and 3.45 ± 1.32, respectively. Compared with group A, the ED of group B was reduced by 24.0%, and the ED of group C was reduced by 22.6%.

### 3.4. Comparison of CT Diagnosis Results with Arthroscopy

The comparison of CT diagnosis results and arthroscopic examination results of 90 patients with knee ACL injury is shown in Table 2. The arthroscopy results were undertaken as the gold standard to judge the diagnosis results. The coincidence rate between CT diagnosis results and arthroscopy diagnosis results was 96.6%. For diagnoses with normal results, the coincidence rate between CT diagnosis results and arthroscopy diagnosis results was 50%, and the coincidence rate between arthroscopy results and CT scan results was 96% (58/60).

## 4. Discussion

ACL injury is a common and frequently occurring disease of knee joint injury, which has a great impact on the normal life of patients [15]. Arthroscopy is currently considered the gold standard for diagnosing ACL injuries. However, arthroscopy is expensive, time-consuming, and invasive. Due to the implementation of arthroscopy, it brings additional pain to a considerable number of cases with negative results of arthroscopy, prolonging the recovery time and increasing the financial burden of the patient [16]. Therefore, early and accurate noninvasive examination has very important clinical significance. Currently, there are three main imaging methods for displaying muscle ligaments, namely, magnetic resonance imaging, ultrasound, and CT [17]. CT has obvious advantages in density and spatial resolution, and the operation is rapid, which provides a high-value imaging basis for the diagnosis and treatment of clinical diseases. However, increased radiation doses may breach the upper limit of the deterministic effect jeopardizing the health of patients. Therefore, how to significantly reduce the radiation dose on the basis of obtaining high-quality image data has become a focus of clinical research. Intelligent algorithms have been widely used in the field of medical image processing [18, 19]. Compared with traditional FBP reconstruction algorithm, CT image iterative reconstruction techniques, such as the iDose4 algorithm, that have appeared in recent years can significantly reduce image noise and improve image contrast and image quality [20–22]. Moreover, there are also relevant materials to study the application of CT in the diagnosis of sports injuries in the recovery of the knee anterior cruciate ligament. Some scholars studied patients with knee trauma and performed dual-source CT or MRI scans. Finally, it was concluded that dual-source CT was safe, rapid, and accurate in diagnosing anterior cruciate ligament injury of the knee joint and can provide a reliable basis for cruciate ligament reconstruction surgery, showing very good application value [23]. CT images of MPR and VRT were processed in patients with ACL injury, and it was found that MPR and VRT images had a positive clinical value in the diagnosis of anterior cruciate ligament injury. Dual-source CT can measure the CT value of the ACL and the thickness of each segment through MPR and VRT postprocessing techniques to diagnose ACL in an objective, quantitative, and noninvasive manner. The degree of ligament injury can be more intuitively predicted using dual-energy staining techniques [24].

The results showed a 96% coincidence rate of CT scan results compared with the gold standard for knee injury examination. It can be explained that CT scanning showed a very good application value in the recovery of knee anterior cruciate ligament sports injury. The results showed that the image quality scores of groups B and C were higher than those of group A, and the comparison between groups B and A was statistically significant. The noise of groups B and C was smaller than that of group A, which was statistically significant (*P* < 0.05). The comparison between groups C and B was also statistically significant (*P* < 0.05). The SNR and CNR of groups B and C were higher than those of group A, but the SNR and CNR of groups B and C were not statistically significant. At the same time, the image noise of patients in group A using the FBP reconstruction algorithm was high, the lesions were difficult to identify, and the boundary was not clear. However, the image noise obtained by the iDose4 reconstruction algorithm in groups B and C was improved, and the image resolution was also higher. The ED of groups A ∼ C were 4.46 ± 1.21, 3.39 ± 1.02, and 3.45 ± 1.32, respectively. Compared with group A, the ED of group B decreased by 24.0%, and the ED of group C decreased by 22.6%. Using the iDose4 reconstruction algorithm in image processing can not only ensure the quality of the image but also reduce the radiation risk to the patient.

## 5. Conclusion

In this study, the application value of CT imaging based on the artificial intelligence reconstruction algorithm in the recovery of knee ACL sports injury was explored. It was found that the image quality obtained by the iDose4 reconstruction algorithm was superior to other algorithms. CT imaging based on artificial intelligence reconstruction algorithm was applied in the diagnosis of ACL sports injury of the knee, the patient's ED was significantly reduced, the radiation dose was reduced by 24.0%, and the result of the CT examination was consistent with that of arthroscopy. Using the iDose4 reconstruction algorithm in image processing had a positive application effect. Nevertheless, the deficiency of this study was that the sample size is too small, which needs further exploration and verification.

## Figures and Tables

**Table 1:** Comparison of general data of all patients.

Group	Gender (male/female)	Age (years)	BMI (kg/m^2^)	Disease site (left knee/right knee)
A (*n* = 30)	18/12	38 ± 4.1	23.34 ± 2.41	20/10
B (*n* = 30)	20/10	39 ± 5.9	23.53 ± 2.73	13/17
C (*n* = 30)	13/17	38 ± 4.6	24.48 ± 1.98	9/21
*P*	0.236	0.672	0.745	0.082

**Table 2:** Comparison of CT diagnosis results and arthroscopy results of all patients.

		CT examination results		
		Damage	Normal	Total
Arthroscopy results	Damage	82	5	87
	Normal	2	1	3
Total		84	6	90

## Data Availability

The data used to support the findings of this study are available from the corresponding author upon request.
